# Preconception consultations with Maternal Fetal Medicine for obese women: a retrospective chart review

**DOI:** 10.1186/s40738-016-0030-9

**Published:** 2017-01-13

**Authors:** Charlotte M. Page, Elizabeth S. Ginsburg, Randi H. Goldman, Chloe A. Zera

**Affiliations:** 1 Harvard Medical School, 25 Shattuck St, Boston, MA 02115 USA; 2 Department of Obstetrics and Gynecology, Brigham and Women’s Hospital, 75 Francis St, Boston, MA 02115 USA

**Keywords:** Obesity, Maternal Fetal Medicine, Preconception consultation, Weight loss

## Abstract

**Background:**

Obesity is associated with impaired fertility and pregnancy complications, and preconception weight loss may improve some of these outcomes. The purpose of this study was to evaluate the quality and effectiveness of Maternal Fetal Medicine (MFM) preconception consults for obese women.

**Methods:**

We performed a retrospective chart review examining 162 consults at an academic medical center from 2008 to 2014. The main outcome measures included consultation content – e.g. discussion of obesity-related pregnancy complications, screening for comorbidities, and referrals for weight loss interventions – and weight loss.

**Results:**

Screening for diabetes and hypertension occurred in 48% and 51% of consults, respectively. Discussion of obesity-related pregnancy complications was documented in 96% of consults. During follow-up (median 11 months), 27% of patients saw a nutritionist, 6% saw a provider for a medically supervised weight loss program, and 6% underwent bariatric surgery. The median weight change was a loss of 0.6% body weight.

**Conclusions:**

In this discovery cohort, a large proportion of MFM preconception consultations lacked appropriate screening for obesity-related comorbidities. While the vast majority of consultations included a discussion of potential pregnancy complications, relatively few patients achieved significant weight loss. More emphasis is needed on weight loss resources and delaying pregnancy to achieve weight loss goals.

## Background

Obesity, defined as a body mass index (BMI) ≥30 kg/m^2^ [1], is the most common health problem in women of reproductive age. Obesity is associated with impaired fertility in women, including decreased rates of pregnancy, higher rates of miscarriage, and decreased rates of live birth with both natural conception and assisted reproductive technology (ART) [2–8]. When pregnancy occurs, obese women are more likely to develop gestational hypertension, preeclampsia, and gestational diabetes [9, 10] and to have a cesarean delivery [11, 12]. Maternal obesity also increases the risk of adverse outcomes in offspring, including congenital anomalies; perinatal, neonatal, and infant death; macrosomia; and childhood obesity [9, 13–15]. There is evidence that reductions in pre-pregnancy BMI reduce the risk of adverse pregnancy outcomes on a population level, although little data on an individual level is available [16, 17]. In addition, a meta-analysis of seven small studies, mostly prospective cohort studies, reported that weight loss in obese women desiring pregnancy increases pregnancy and live birth rates [18]. However, a recent randomized controlled trial found that a six-month lifestyle intervention program made no difference in live birth rates for obese infertile women undergoing infertility treatment [19].

In light of the adverse effects of obesity and potential benefits of weight loss, some obese women are referred to Maternal Fetal Medicine (MFM) for a preconception consultation. Ideally, an MFM consult should not only inform an obese woman of the impact of her weight on fertility and pregnancy, but also equip her with strategies for weight loss. To our knowledge, preconception MFM consultations for obese women have not been previously studied. We therefore sought to evaluate the quality of obesity management in preconception MFM consults in our center, examining both documented content and weight loss outcomes.

## Methods

We performed a retrospective chart review to evaluate MFM preconception consultations for obese women at Brigham & Women’s Hospital (BWH) between January 1, 2008 and December 31, 2014. From a database of consult referrals, we identified all subjects who were referred for obesity, hypertension, and/or diabetes mellitus. Of these patients, we included all those who were obese (BMI ≥30 kg/m^2^) and were not pregnant at the time of initial consultation. We excluded patients if they did not have at least one follow-up visit with Obstetrics & Gynecology during the study period. If a patient had more than one MFM consult during the study period, we only included the most recent consult. We reviewed medical records through December 31, 2015 for weight, fertility, and pregnancy outcomes.

If the MFM or referring provider identified diabetes, hypertension, prior bariatric surgery, and/or polycystic ovary syndrome as an existing problem, we reported it as a comorbidity. We considered diabetes screening ‘done’ if the patient was known to have diabetes at the time of MFM consult or if the MFM provider reported a recent screen or plans to perform a new screen. If the patient had a known diagnosis of hypertension or documentation of a blood pressure in the MFM consultation note, we considered hypertension screening ‘done.’ We considered discussion of obesity-related pregnancy complications to have occurred if the MFM note stated that this subject was discussed. We considered discussion of diet, physical activity, and bariatric surgery to have occurred if the MFM note mentioned these topics.

We designated referrals to nutrition, a medically supervised weight loss program, and bariatric surgery as having occurred if the patient had already seen the referred service, the patient had already been offered referral by another provider, the MFM note reported offering referral, or there was a note from the referred service documenting referral. The medically supervised weight loss program referred specifically to BWH’s hospital-based Program for Weight Management, which includes calorie-controlled diet and liquid diet programs in addition to other medical treatments for obesity. Per protocol since 2010, all women undergoing evaluation for infertility in the Reproductive Endocrinology & Infertility (REI) Division at BWH with BMI >40 should be referred to the Program for Weight Management. We considered nutrition consultation and bariatric surgery to have occurred, potentially outside of BWH, if they were documented in the medical record.

The follow-up period was the time between the MFM consult and the last encounter with Obstetrics & Gynecology during the study period (ending December 31, 2015) or conception of an ongoing pregnancy, whichever came first. We defined pregnancy as any pregnancy during the follow-up period, including chemical pregnancies. Ongoing pregnancy was defined as an intrauterine pregnancy that resulted in live birth during the study period or was at or beyond 10 weeks of gestation at the end of the study period. We measured time to achieve an ongoing pregnancy from the date of the MFM consult.

Fertility treatments included intrauterine insemination (IUI), in vitro fertilization (IVF), or use of any of the following medications: metformin in a patient without diabetes, bromocriptine, cabergoline, clomiphene citrate, follicle-stimulating hormone, gonadotropin-releasing hormone, human chorionic gonadotropin, human menopausal gonadotropin, letrozole, anastrozole, leuprolide, nafarelin acetate, or goserelin acetate. We designated participants as starting fertility treatment if their record indicated use of treatments either prior to MFM consultation – if they were referred to MFM by the treating provider – or during follow-up.

Baseline weight was either the weight measured at the time of MFM consult or the most recent weight within the prior three months. Final weight was the weight at the first prenatal visit or at an appointment during the last fertility treatment cycle. For women who underwent bariatric surgery during the study period, we excluded any weights after surgery to avoid misrepresenting summary statistics for typical weight changes. We stratified subjects into three groups by BMI: 30.0 − 39.9, 40.0 − 49.9, and ≥50.0 kg/m^2^.

As appropriate, we presented descriptive results as frequencies with percentages or as medians with interquartile ranges. We compared categorical variables among BMI groups using chi-square tests or Fisher’s exact tests, while we used Kruskal-Wallis tests for continuous variables. Using logistic regression, we calculated odds ratios for any pregnancy and for ongoing pregnancy, adjusted for age. To determine the impact of obesity class on pregnancy rates, we used the group with BMI 30.0 − 39.9 kg/m^2^ as the referent group to which each of the two higher categories was compared. For all statistical tests, we considered a two-sided *p*-value ≤0.05 to be significant. We performed all analyses using STATA/SE 12.1 (StataCorp LP, College Station, TX). The BWH Institutional Review Board approved this study, including a waiver of informed consent (protocol # 2015P000843, approved 5/6/15).

## Results

**Fig. 1:**
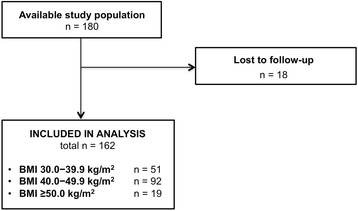
Flow diagram of study participants. Available study population was all obese patients (BMI ≥30 kg/m^2^) identified from our referral database who were seen by Maternal Fetal Medicine at Brigham & Women’s Hospital for preconception consultation in 2008–2014. Abbreviations: BMI = body mass index

As the flow diagram of study participants in Fig. 1 indicates, we identified 180 patients with a BMI ≥30 kg/m^2^ who underwent MFM consultation. Of these, we excluded 18 because they were lost to follow-up, leaving 162 participants in the final analyses. Table 1 presents the characteristics of women at the time of MFM consult, stratified by BMI. There were 51 subjects with BMI 30.0 − 39.9 kg/m^2^, 92 with BMI 40.0 − 49.9 kg/m^2^, and 19 with BMI ≥50.0 kg/m^2^. Distribution of BMI differed by race and by referral source. There were more white women in the middle BMI category (64%) than in the lower (59%) or upper categories (53%; *p* = 0.02). In addition, there were more women referred by REI in the middle category (97%) than in the lower (86%) or upper categories (89%); *p* = 0.04.

**Table 1:** Characteristics of participants at the time of Maternal Fetal Medicine consultation, by body mass index^a^

Characteristic	BMI 30.0 − 39.9	BMI 40.0 − 49.9	BMI ≥50.0	*p*-value
	*n* = 51	*n* = 92	*n* = 19	
Age, in years	36 (33–40)	36 (33–40)	37 (33–41)	0.61
Gravidity	0 (0–1)	1 (0–1)	0 (0–1)	0.31
Parity	0 (0–0)	0 (0–0)	0 (0–0)	0.98
Race/ethnicity				0.02
Non-Hispanic, non-Latino white	30 (59%)	59 (64%)	10 (53%)	
Black/African American	11 (22%)	10 (11%)	6 (32%)	
Hispanic/Latino	1 (2%)	13 (14%)	0 (0%)	
Unknown/other	9 (18%)	10 (11%)	3 (16%)	
Insurance type				0.92
Public	5 (10%)	7 (8%)	1 (5%)	
Private	46 (90%)	85 (93%)	18 (95%)	
Referral source				0.04
REI	44 (86%)	89 (97%)	17 (89%)	
Other	7 (14%)	3 (3%)	2 (11%)	
Comorbidities				
Diabetes	11 (22%)	8 (9%)	3 (16%)	0.08
Hypertension	19 (37%)	24 (26%)	5 (26%)	0.36
Prior bariatric surgery	7 (14%)	10 (11%)	0 (0%)	0.22
PCOS	17 (37%)	26 (28%)	3 (16%)	0.23

*Abbreviations*: *BMI* body mass index, in kg/m^2^, *REI* Reproductive Endocrinology & Infertility, *PCOS* polycystic ovary syndrome

^a^Data expressed as median (interquartile range) or frequency (%). Categorical variables were compared among BMI groups using chi-square tests or Fisher’s exact tests, while continuous variables were compared using Kruskal-Wallis tests

Table 2 shows data on documented consult content, including comorbidities screening, discussion topics, and referrals. Screening for diabetes and hypertension each occurred in approximately half of MFM consults (48% and 51%, respectively), as did documented discussion of diet and physical activity (57% and 56%, respectively). Discussion of obesity-related pregnancy complications was documented for 96% of the consults. The frequency of screening for hypertension differed by BMI, with a lower rate of screening in the middle category (42%) than in the lowest (63%) and highest categories (63%); *p* = 0.04. Documented discussion of obesity-related pregnancy complications, diet, physical activity, and bariatric surgery – as well as referrals to weight loss interventions – were all most common in the highest BMI group. BMI was significantly associated with discussion of obesity-related pregnancy complications, discussion of physical activity, nutrition referral, and referral to a medically supervised weight loss program (*p* = 0.02, 0.04, 0.006, and 0.001, respectively); however, the associations of BMI with discussion of diet, discussion of bariatric surgery, and referral to bariatric surgery were not significant. The likelihood of nutrition referral in the highest BMI group was more than double that in the lowest BMI group (58% vs. 22%, respectively; *p* = 0.006).

**Table 2:** Documented content in Maternal Fetal Medicine consultations for obese women, by body mass index^a^

Consult feature	Overall	BMI 30.0–39.9	BMI 40.0–49.9	BMI ≥50.0	*p*-value^b^
	*N* = 162	*n* = 51	*n* = 92	*n* = 19	
Diabetes screen	78 (48%)	25 (49%)	42 (46%)	11 (58%)	0.62
Hypertension screen	83 (51%)	32 (63%)	39 (42%)	12 (63%)	0.04
Discussion of obesity-related pregnancy complications	156 (96%)	46 (90%)	91 (99%)	19 (100%)	0.02
Discussion of diet	92 (57%)	25 (49%)	52 (57%)	15 (79%)	0.08
Discussion of physical activity	91 (56%)	31 (61%)	45 (49%)	15 (79%)	0.04
Discussion of bariatric surgery	63 (39%)	14 (27%)	39 (42%)	10 (53%)	0.09
Referral to nutrition	62 (38%)	11 (22%)	40 (43%)	11 (58%)	0.006
Referral to medically supervised weight loss program	38 (23%)	3 (6%)	29 (32%)	6 (32%)	0.001
Referral to bariatric surgery	57 (35%)	12 (24%)	36 (39%)	9 (47%)	0.09

*Abbreviations*: *BMI* body mass index, in kg/m^2^

^a^Data expressed as frequency (%). Comparisons among BMI groups employed chi-square tests or Fisher’s exact tests, as appropriate

^b^Comparing the three BMI categories

Table 3 presents comparisons of follow-up time and referral outcomes among groups. The median follow-up time was 11 months, and the mean was 14 ± 14 months. Twenty-seven percent of subjects saw a nutritionist (69% of those referred). Meanwhile, 24% of those referred to the medically supervised weight loss program had a visit, constituting 6% of all subjects. Likewise, 6% of participants underwent bariatric surgery during the study period.

**Table 3:** Outcomes following Maternal Fetal Medicine consultations for obese women, by baseline body mass index^a^

Outcome	Overall	BMI 30.0–39.9	BMI 40.0–49.9	BMI ≥50.0	*p*-value^b^
	*N* = 162	*n* = 51	*n* = 92	*n* = 19	
Follow-up time, in months	11 (4–21)	12 (3–22)	10 (4–22)	10 (4–15)	0.91
Saw nutritionist	43 (27%)	8 (16%)	28 (30%)	7 (37%)	0.09
Saw provider for medically supervised weight loss program	9 (6%)	1 (2%)	8 (9%)	0 (0%)	0.16
Underwent bariatric surgery during study period	10 (6%)	0 (0%)	8 (9%)	2 (11%)	0.05
Started fertility treatment	119 (74%)	35 (69%)	72 (78%)	12 (63%)	0.26
Time after consult until starting treatment, in days	30 (8–79)	23 (−2–85)	33 (13–99)	21 (2–48)	0.40
Underwent IVF	102 (63%)	31 (61%)	60 (65%)	11 (58%)	0.77
Underwent IUI	24 (15%)	11 (22%)	10 (11%)	3 (16%)	0.21
Achieved any pregnancy	104 (64%)	29 (57%)	68 (74%)	7 (37%)	0.004
Achieved ongoing pregnancy	82 (51%)	24 (47%)	53 (58%)	5 (26%)	0.04
Time to achieve ongoing pregnancy, in months	9 (3–15)	11 (3–16)	9 (3–16)	10 (4–11)	0.61

*Abbreviations*: *BMI* body mass index, in kg/m^2^, *IVF* in vitro fertilization, *IUI* intrauterine insemination

^a^Data expressed as median (interquartile range) or frequency (%). Categorical variables were compared among BMI groups using chi-square tests or Fisher’s exact tests, while continuous variables were compared using Kruskal-Wallis tests

^b^Comparing the three BMI categories

Data on follow-up weights, available for 129 of the 162 participants, appear in Table 4. The median weight change was a loss of 2.0 lb, or 0.6% body weight, over a median of 12 months. Of the 129 participants with follow-up weights, 25 (19%) achieved ≥5% loss and 7 (5%) achieved ≥10% loss. Weight loss was associated with follow-up time; women who lost ≥5% body weight had a median of 18 months between baseline and final weights, whereas those who did not reach that target had a median of 10 months between weights (*p* = 0.006). Weight change was not associated with whether the MFM consult occurred before 2010, when the REI Division began mandating that women with BMI >40 be referred to the Program for Weight Management. The median weight change was a gain of 1.0 lb for the 22 participants with MFM consults prior to 2010 and a loss of 2.0 lb for the 107 participants with consults in 2010 or later (*p* = 0.89).

**Table 4:** Weight outcomes after Maternal Fetal Medicine consultations for obese women, by baseline body mass index^a^

Outcome	Overall	BMI 30.0–39.9	BMI 40.0–49.9	BMI ≥50.0	*p*-value^b^
	*n* = 129	*n* = 40	*n* = 77	*n* = 12	
Time between baseline and final weights, in months	12 (5–19)	13 (5–20)	11 (5–21)	12 (7–15)	0.74
Weight change, in pounds	−2.0 (−10.5–5.5)	0.5 (−5.0–8.0)	−2.0 (−11.5–4.5)	−5.0 (−11.5–6.0)	0.41
Weight change, as % body weight	−0.6 (−3.6–2.2)	0.3 (−2.4–3.6)	−0.7 (−4.5–1.9)	−1.7 (−3.5–1.9)	0.43
Lost ≥5% body weight	25 (19%)	7 (18%)	17 (22%)	1 (8%)	0.56

*Abbreviations*: *BMI* body mass index, in kg/m^2^

^a^Includes the 129 subjects with follow-up weights. Data expressed as median (interquartile range) or frequency (%). Categorical variables were compared among BMI groups using chi-square tests or Fisher’s exact tests, while continuous variables were compared using Kruskal-Wallis tests

^b^Comparing the three BMI categories

Fertility outcomes stratified by BMI groups are included in Table 3. The majority of participants (76%) started fertility treatment 30 days after MFM consultation. Of the women who started fertility treatment, 86% underwent IVF and 20% underwent IUI. Crude pregnancy rates, including both spontaneous conceptions and conceptions using fertility treatment, differed significantly across BMI categories, with the lower two categories having higher rates (57% and 74%, respectively) than the highest BMI category (37%). Rates of ongoing pregnancy (excluding chemical pregnancies and miscarriages) followed a similar pattern. Thirteen percent of ongoing pregnancies were spontaneous conceptions, while 87% were achieved using fertility treatment.

In age-adjusted logistic regression with BMI 30.0–39.9 kg/m^2^ as the referent group, the rate of any pregnancy for women with BMI 40.0–49.9 kg/m^2^ was significantly higher; the odds ratio was 2.15 (95% CI 1.04–4.43). However, odds ratios comparing women in this BMI group to those in the referent group for rates of ongoing pregnancy were not significant, nor were odds ratios for pregnancy rates comparing women with BMI ≥ 50.0 kg/m^2^ to the referent group. Rates of any pregnancy and of ongoing pregnancy were not associated with whether women lost ≥5% body weight (*p* = 0.33 and 0.74, respectively).

## Discussion

Research on the impact of MFM consults on obesity is lacking. To our knowledge, no prior studies on this topic have been published (PubMed; 1950-December 2016; English language; search terms “obesity,” “preconception,” and “consult”). In the present study, we conducted a retrospective review of MFM consultations for obesity in our institution. Nearly all of these consultations included a discussion of pregnancy complications associated with obesity; however, the consults were unsuccessful in meaningfully effecting pre-pregnancy weight loss. In this study, only 19% of the participants with follow-up weights achieved ≥5% loss, and only 5% achieved ≥10% loss. We believe that increased emphasis is needed on weight loss resources, including discussion of lifestyle modification and referrals to specialty obesity treatment services, e.g. bariatric surgery. In addition, MFM providers and referring REI providers must be allied in counseling women to delay fertility treatment and conception to focus on weight loss. This recommendation is more nuanced in the case of women of advanced maternal age, when postponing fertility treatment may result in loss of the fertile window and may therefore be untenable.

Increased attention is needed in MFM consultations to baseline hypertension and diabetes screening, as these are underlying disorders that should ideally be controlled prior to attempts at conception. Obesity is associated with increased risk of these comorbidities, and preconception care for diabetic women significantly improves pregnancy outcomes, making preconception diagnosis superior to diagnosis during pregnancy [20]. Rates of hypertension screening in our study were particularly low in women with BMI 40.0–49.9 kg/m^2^, perhaps because the patients in the lowest BMI group had a higher prevalence of hypertension (see Table 1) and those in the highest group had greater perceived risk.

Guidelines from the American College of Obstetricians and Gynecologists recommend counseling women on weight loss and referring them to weight loss services prior to conception [15]. Although there is no evidence-based strategy for preconception weight loss [21], it is generally accepted that lifestyle interventions combining diet, exercise, and behavioral therapy should be employed for all obese individuals [22]. In our study, discussion of physical activity and referrals to a nutritionist and a medically supervised weight loss program were significantly more common in higher BMI groups, suggesting a lost opportunity to optimize outcomes in women with less weight to lose.

Bariatric surgery has been shown to result in more significant and persistent weight loss than lifestyle modification alone [23], and it is associated with lower risk of obesity-associated pregnancy complications [24, 25]. Bariatric surgery is indicated for women with BMI ≥40 kg/m^2^ or women with BMI ≥35 kg/m^2^ and a comorbidity [22]. It has been recommended that women wait 12–18 months after surgery before conceiving [24], which may deter infertile women from pursuing this weight loss method, particularly if they are over age 35. The median age in our study was 36 years old, which may explain why less than 40% of women were referred to a bariatric surgeon.

Advanced maternal age might have been a significant contributing factor to the overall lack of pre-pregnancy weight loss in our study population since older women may be unlikely to delay attempts at pregnancy to work on weight loss. Indeed, the majority of women seen for MFM consultations in our study started fertility treatment, typically within one month, and the number of women referred to a medically supervised weight loss program who actually attended was low. These statistics suggest that patients’ priorities are more focused on becoming mothers than on managing their own obesity. Moreover, the short interval between MFM consults and the initiation of fertility treatment confirms that REI physicians in our institution do not require weight loss before conception attempts. In contrast to the low attendance rate for referrals to a medically supervised weight loss program, the attendance rate for those referred to nutrition was quite high, perhaps because this intervention is perceived as a smaller time commitment and/or because insurance companies in Massachusetts often require it in order to pay for ART.

The conclusions of our study are limited by its retrospective nature, small size, and location at a single medical center. Due to its small sample size, our study lacked power to detect differences in certain variables across BMI groups. For example, in our logistic regression analysis, the power to detect a difference in crude pregnancy rates between the BMI 30.0–39.9 kg/m^2^ and BMI ≥50 kg/m^2^ groups, based on the observed sample sizes and rates, was 33%. Another limitation was our study’s retrospective chart review design, which required relying on perfect documentation of consult content by the MFM specialist. Also as a result of the retrospective design, we did not have access to a comparison group of women planning pregnancy who were obese and did not receive MFM consultation. Therefore, we cannot draw definitive conclusions about the impact of an MFM consult on obese women’s weight or fertility. Finally, this study was performed at a single institution, limiting the generalizability to different patient populations and MFM departments.

## Conclusions

Our study has value as the first to examine preconception MFM consults for obesity. In our cohort, a large proportion of MFM preconception consultations lacked appropriate screening for obesity-related comorbidities. While the vast majority of consultations included a discussion of potential pregnancy complications, relatively few patients achieved significant weight loss. In light of obesity’s adverse effects on fertility and pregnancy, the preconception consult is a critical opportunity for intervention. To make the most of this opportunity, MFM providers should counsel every obese patient about weight loss strategies and offer referrals to appropriate services. They should provide specific recommendations on diet and exercise, including caloric reduction and frequency and intensity of physical activity. Commitment on the part of REI physicians and patients to delay fertility treatment to focus on weight loss is also needed, with exceptions for those women of advanced maternal age for whom postponing fertility treatment might lead to an unacceptable decline in fertility. If these practices became standard, more obese women seen for preconception MFM consultation might achieve meaningful weight loss.

## Abbreviations

ART: Assisted reproductive technology
BMI: Body mass index
BWH: Brigham & Women’s Hospital
CI: Confidence interval
IUI: Intrauterine insemination
IVF: In vitro fertilization
MFM: Maternal Fetal Medicine
REI: Reproductive Endocrinology and Infertility
